# Opposite Genetic Effects of *CMIP* Polymorphisms on the Risk of Type 2 Diabetes and Obesity: A Family-Based Study in China

**DOI:** 10.3390/ijms19041011

**Published:** 2018-03-28

**Authors:** Yaying Cao, Tao Wang, Yiqun Wu, Juan Juan, Xueying Qin, Xun Tang, Tao Wu, Yonghua Hu

**Affiliations:** 1 Department of Epidemiology and Biostatistics, School of Public Health, Peking University, Beijing 100191, China; cyyenjoy@126.com (Y.C.); qywu118@163.com (Y.W.); juanjuan@bjmu.edu.cn (J.J.); xueyingqin@gmail.com (X.Q.); tangxun@bjmu.edu.cn (X.T.); twu@bjmu.edu.cn (T.W.); 2 Department of Epidemiology and Population Health, Albert Einstein College of Medicine, Bronx, NY 10461, USA; tao.wang@einstein.yu.edu

**Keywords:** pleiotropic genetic effects, *CMIP*, type 2 diabetes mellitus, obesity, sex-specific pattern, family-based study

## Abstract

C-Maf Inducing Protein (*CMIP*) gene polymorphisms were reported to be associated with type 2 diabetes mellitus (T2DM). Whether the association between *CMIP* and T2DM is mediated via obesity-related phenotypes is still unclear. We analyzed the association of *CMIP* rs2925979 with T2DM and a comprehensive set of obesity-related phenotypes in 1576 families ascertained from a Chinese population. These families included a total of 3444 siblings (1582 with T2DM, 963 with prediabetes, and 899 with a normal glucose level). Using multi-level mixed effects regression models, we found that each copy of *CMIP* rs2925979_T allele was associated with a 29% higher risk of T2DM in females (*p* = 9.30 × 10^−4^), while it was not significantly associated with T2DM in males (*p* = 0.705). Meanwhile, rs2925979_T allele was associated with lower levels of body mass index (BMI), waist circumference (WC), hip circumference (HC), percentage of body fat (PBF), PBF of arms, PBF of legs, and PBF of trunk in nondiabetes females (all *p* < 0.05). The opposite associations of rs2925979_T allele with T2DM and obesity-related phenotypes suggest that *CMIP* may exert independent pleiotropic effects on T2DM and obesity-related phenotypes in females.

## 1. Introduction

Both type 2 diabetes mellitus (T2DM) and obesity are challenging pandemics worldwide [1]. Epidemiological data suggested that obesity, especially central obesity, was positively associated with T2DM risk [2,3,4]. Shared genetic influences on T2DM and obesity-related phenotypes were found in family studies [5,6]. Genome-wide association studies (GWAS) have established at least 75 susceptibility loci of T2DM [7], and many of these T2DM-loci were associated with obesity-related phenotypes. However, the associations of these T2DM-loci were highly heterogeneous, even opposite, for different obesity-related phenotypes [1], suggesting that the genetic link between various obesity-related phenotypes is complicated.

Epidemiological studies showed significant gender differences for both adiposity distribution [8,9] and T2DM [10,11]. For example, males tend to accumulate more visceral and hepatic adipose tissue, while females accumulate more peripheral and subcutaneous adipose tissue [8,12]. T2DM is more frequently diagnosed in younger male patients with a lower body mass index (BMI) than in females. In addition to differences in culture, lifestyle, environment, and sex hormones between males and females [9], genetic sexual dimorphism may be another reason for this differential diagnosis. For central obesity phenotypes, genetic variants often have a larger genetic effect in females than in males [13,14,15,16].

*CMIP* (C-Maf-Inducing Protein) gene is a protein-coding gene located on 16q23.2-q23.3 and involved in multiple signaling pathways related to obesity and T2DM, such as nuclear factor-κB (NF-κB) signaling pathway [17] and T-helper 2 (Th2) signaling pathway [18]. Previously, rs2925979 in this gene region was reported to be associated with an increased risk of T2DM in a European population [19] and a population with multi-ancestry [20]. In addition, rs2925979 was found to be associated with waist-to-hip ratio adjusted for body mass index (WHRadjBMI) in European and East Asian females [21,22]. However, it is still not clear how rs2925979 is associated with other obesity-related phenotypes, and whether its association with T2DM is mediated by obesity-related phenotypes.

In this paper, we analyzed data of 1576 families ascertained from a Chinese population to examine the association of the *CMIP* locus with T2DM and with a comprehensive set of obesity-related phenotypes. Given the known sexual dimorphism of obesity-related phenotypes, we studied the possible sex–gene interactions.

## 2. Results

In total, we recruited 3444 siblings (aged ≥40 years) from 1576 families. Among these participants, 1582 were T2DM patients and 1862 were nondiabetic. In nondiabetic individuals, there were 963 prediabetes subjects and 899 subjects with a normal glucose level (termed as “normal” in the following context), respectively. The average age of all participants was 58.9 years old, and 48.8% of them were males. The frequencies of rs2925979_T allele were 0.41 for all the participants and 0.39, 0.39, and 0.42 for normal participants, prediabetes, and T2DM patients, respectively. No difference was found in the frequency of rs2925979_T allele between genders (0.40 for males and 0.41 for females; *p* = 0.740). Table 1 shows the characteristics of the enrolled participants according to the T2DM status. As expected, the subjects in the three groups differed in age, sex, hypertension, hyperlipidemia, smoking status, and alcohol drinking (*p* < 0.05). The TT genotype frequency in T2DM subjects was higher in comparison to the other two groups, although it was not significant.

We first compared the levels of obesity-related phenotypes between the normal group and the prediabetes group, excluding T2DM patients because of possible reversal causality. Table 2 shows that BMI, waist circumference (WC), percentage of body fat (PBF), PBF of arms, PBF of legs, and PBF of trunk were higher in prediabetes than in normal subjects for both genders (*p* < 0.05). Hip circumference (HC) and waist-to-hip ratio (WHR) were higher in prediabetes males than in normal males (*p* < 0.05). Most of these associations remained significant after Bonferroni correction (*p* < 0.006). WHR-adjusted BMI (WHRadjBMI) was not significantly different between the two groups in either gender (*p* > 0.05).

We then examined the association between rs2925979 and T2DM (T2DM versus nondiabetes). For all participants, each copy of rs2925979_T allele was associated with a 15% higher risk of T2DM (odds ratio (*OR*): 1.15, 95% confidence interval (*CI*): 1.02–1.30, *p* = 0.022) (Table 3, Model 1). In the sex-stratified analysis, each copy of rs2925979_T allele was associated with a 29% higher risk of T2DM in females (*OR*: 1.29, 95% *CI*: 1.11–1.50, *p* = 9.30 × 10^−4^), but not in males (*OR*: 0.96, 95% *CI*: 0.79–1.17, *p* = 0.705). There was a significant sex–single nucleotide polymorphism (SNP) interaction (*p*_interaction_ = 0.021) (Table 3, Model 1). The results were similar when adjusted for age, hypertension, hyperlipidemia, smoking status, and alcohol drinking (Table 3, Model 2).

Before examining the associations of rs2925979 with multiple obesity-related phenotypes, we checked the correlations among different obesity-related phenotypes in nondiabetes (Supplementary Table S1). The values of the Pearson correlation coefficients ranged from −0.179 to 0.983. Table 4 shows the results of the association analysis between rs2925979 and obesity-related phenotypes among nondiabetic subjects. Rs2925979_T allele was negatively associated with BMI, WC, HC, and PBFs in nondiabetes females (*p* < 0.05), and most of these associations remained significant after Bonferroni correction (*p* < 0.006). However, no significant association with any obesity-related phenotype was found in nondiabetes males (*p* > 0.05). Significant SNP–gender interactions were found in BMI, HC, PBF, PBF of arms, and PBF of legs (*p* < 0.05), while these interactions disappeared after Bonferroni correction (*p* > 0.006).

We also conducted an association analysis by categorizing the nondiabetes females according to quartiles of obesity-related phenotypes to avoid the assumption of a linear trend in the associations (Supplementary Figure S1). The effect sizes of rs2925979_T allele on most obesity-related phenotypes appeared to be increasing with the rising gradients of quartiles. The associations of rs2925979_T allele with BMI, WC, HC, and PBF of trunk were significant among nondiabetes females in the Quartile 4 of the corresponding phenotypes (*p* < 0.05).

To examine the impact of T2DM on the associations between rs2925979 and obesity-related phenotypes, we also conducted a similar analysis among T2DM patients (Supplementary Table S2). In contradiction to the results in nondiabetes subjects, the associations with most obesity phenotypes in female T2DM patients disappeared, except for HC (*p* < 0.05). Interestingly, rs2925979_T allele was positively associated with WHR in male T2DM patients and with WHRadjBMI in both male and female T2DM patients (*p* < 0.05). The significance of the associations between obesity-related phenotypes and rs2925979 among male and female T2DM patients, respectively, disappeared after Bonferroni correction (*p* > 0.006).

## 3. Discussion

Our result revealed that *CMIP* rs2925979_T allele was associated with T2DM risk in a sex-specific pattern: the positive association of rs2925979_T allele with T2DM risk was observed in females, but not in males. The sex–gene interaction was significant. Previously, rs2925979_T allele was found to be associated with a higher T2DM risk, but without accounting for sex heterogeneity [19,20]. The heterogeneous effect of rs2925979 depending on gender for T2DM reported in the current study is a novel finding. The molecular mechanism underlying this sex-specific association remains unclear. However, previous studies revealed heterogeneous associations by gender with T2DM for other loci, with effects present only in one gender, or even manifesting in opposite directions in the two genders. For example, evidence for the linkage of Chromosomes 2 and 5 with T2DM was found in females only [23]. Female-specific associations with T2DM were demonstrated for multiple genes, such as *CDKN2A/2B*, *KCNJ11*, and *TCF7L2* [24,25,26,27,28,29,30]. Male-specific associations with T2DM also involved various genes, such as *TCF7L2*, *ELMO1*, and *BCL11A* [31,32,33,34]. Furthermore, the minor allele of rs4235308 was associated with a higher risk of T2DM in females, while it played a protective role for T2DM in males [35]. Because of the existence of differential genetic associations of various loci with T2DM depending on gender, as revealed by previous studies, it is necessary to perform association analyses for T2DM in a sex-stratified way to understand potentially different mechanisms of T2DM in the two genders. The novel female-specific genetic association with T2DM found in our study may contribute to identify new mechanisms or pathways of T2DM, unique or more active in females. For genetic variants in autosomal chromosomes, research suggested that sex could be regarded as an “environmental” factor [36]. *CMIP* rs2925979 is an intron variant located on chromosome 16. Studies are warranted to understand the mechanisms of the sex-specific genetic effect of rs2925979 on T2DM, in terms of cellular, anatomical, physical, and metabolic differences between genders.

In the present study, we also explored the association of rs2925979 with multiple obesity-related phenotypes. BMI is often considered as a measure of overall obesity, and WC, HC, and PBFs reflect fat accumulation in corresponding parts of body, while WHR and WHRadjBMI represent fat accumulation in the abdomen. In the current study, rs2925979_T allele was nominally associated with lower levels of all obesity-related phenotypes in nondiabetes females, except for WHR and WHRadjBMI. This result implies that, in females, rs2925979_T allele was negatively associated with fat accumulation all over the body, rather than exclusively in the abdomen. Previously, rs2925979_T allele was found to be positively associated with spontaneous lipolysis [37]. Rs2925979_T allele may alleviate fat accumulation through enhancing the lipolysis pathway, providing an explanation for the negative associations with obesity-related phenotypes found in our study.

Similar to the exclusive genetic effects on obesity in females reported in our study, previous sex-specific GWAS studies of obesity also showed sexual dimorphism [13,14,15,16,38], often with a larger genetic effect in females than in males. The sexual dimorphism was mainly found for central obesity phenotypes, while evidence of sex-specific genetic effects on BMI is still limited. Intriguingly, in the current study, rs2925979_T allele was negatively associated with BMI in females, whileit was not associated with central obesity phenotypes as WHR and WHRadjBMI. Previously, rs2911280, located in an intron of *CMIP*, was found to be associated with dihydroepiandrosterone sulphate (DHEAS), a precursor to androgens and estrogens [39]. As an intron variant of *CMIP*, rs2925979 may also have the potential of being associated with sex hormones in obesity, and studies are needed to verify this hypothesis. Previous GWAS studies reported a positive association of rs2925979_T allele with WHRadjBMI in females [21,22]. We did not find such an association in nondiabetes females. However, we found nominally significant associations in both male and female T2DM patients. This result suggests that the association of rs2925979 with WHRadjBMI reported before might have been influenced by T2DM and hypoglycemic drug use.

It is well known that accumulation of adipose tissue is closely associated with the development of insulin resistance in peripheral tissues and with a chronic low-grade inflammatory state, thus increasing T2DM risk [40]. In our study, the levels of most obesity-related phenotypes were also higher in the prediabetes group than in the normal group. However, our study showed that rs2925979_T allele was associated with an increasing risk of T2DM but with lower levels of most obesity-related phenotypes. In fact, similar opposite effects on obesity and T2DM were observed for other variants in previous studies. For example, the T2DM-increasing allele of *ARL15,* rs702634, was reported to be associated with a lower BMI level [41]. In addition, the BMI-increasing allele of *QPCTL*, rs2287019, and the WHR-increasing allele rs4846567 were both associated with increased insulin sensitivity [42]. The BMI-increasing alleles rs2287019 and rs713586 were associated with a lower 2 h glucose level, and the WHR-increasing allele of *CPEB4,* rs6861681, was associated with a lower fasting glucose level [43]. The reason that one single genetic variant was associated with a better glucose metabolism and a higher level of obesity, or vice versa, is not clear. Adipose tissue is considered as an important endocrine organ secreting adipokines, and previous studies showed that a higher level of obesity is associated with a higher risk of cancer [44], cardiovascular diseases, and mortality [45]. The phenomena that one single genetic variant being associated with both a good metabolic outcome and a bad one simultaneously may be the result of balancing selection [46].

The strengths of the present study include the family-based design. Environmental exposures among family members are more likely to be similar, thus reducing the noise from environmental factors. Another strength is that we examined multiple phenotypes of obesity, making it possible to identify distinct effects on different obesity-related phenotypes. The sex-specific analyses also provided an opportunity to understand the role that sex plays in the mechanisms of both T2DM and obesity. The limitations include that the participants in our study were recruited from a local community from Northern China, and the results may not be generalizable to other populations. The sample size was relatively small for sex–SNP interactions. We cannot exclude potential false negative results.

## 4. Materials and Methods

### 4.1. Study Population

Our sib-pair study was conducted in Fangshan, a rural district located in Beijing, China. We recruited full siblings aged ≥40 years, while step siblings or adopted siblings were not included. Participants were divided into a T2DM group and a nondiabetes group. People with nondiabetes were further classified into a prediabetes group and a normal group (having with normal glucose levels). A total of 3444 participants were included in the current study.

This study has been approved by the Ethics Committee of Peking University Health Science Center (IRB00001052-13027), Beijing, China (22 July 2013). Written informed consent was obtained from every participant. The study was conducted in accordance with the Declaration of Helsinki.

### 4.2. Ascertainment of T2DM Status, Including T2DM, Prediabetes, and Normal Glucose Levels

The status of T2DM was defined according to the American Diabetes Association criteria as commonly done [2]. Detailed inclusion and exclusion criteria for enrollment are presented in Supplementary Table S3.

### 4.3. Measurement and Calculation of Obesity-Related Phenotypes

Height was measured without shoes by a fixed stadiometer, and weight was measured without shoes or heavy clothes by a traditional scale. The default value of tare weight was set as 1 kg. WC and HC were measured horizontally with people standing relaxed and in light clothes. WC was measured at the midpoint between the lower costal margin and the iliac crest, while HC was measured at the level of maximum extension of the buttocks. In addition to PBF, we also obtained fat mass, lean body mass, and muscle mass for five segments of the body (right arm, left arm, trunk, right leg, and left leg), respectively, by using Tanita BC-418 body composition analyzer (Tanita, Tokyo, Japan).

BMI was calculated as weight (kg)/(height (m))^2^, and WHR was calculated as WC (cm)/HC (cm). WHRadjBMI was calculated as the residual of WHR adjusted for age, age^2^, and BMI, for men and women separately, and then transformed by the inverse standard normal function [21]. PBF of arms, legs, and trunk was calculated as fat mass (kg)/weight (kg) × 100% for the corresponding parts of the body.

### 4.4. Genotyping

DNA was extracted from venous blood samples. We performed DNA genotyping using MassARRAY iPLEX platform (Sequenom Inc., San Diego, CA, USA), following the manufacturer’s protocol. We assessed SNP genotypes by MassARRAY Typer Analyzer software (v.4.0, Sequenom Inc., San Diego, CA, USA). The call rate for rs2925979 was above 95%. To verify reproducibility, blinded duplicates were selected randomly and run along with the samples. For Hardy–Weinberg equilibrium test, we randomly selected one sibling without T2DM from each family and found no violation for rs2925979 (*p* > 0.001).

### 4.5. Assessment of Covariates

Anthropometric characteristics were collected in person through a structured questionnaire modified from the PhenX Toolkit [47], collecting information about pedigree number, relationship with other participants, demographic characteristics, disease history, family history, medication use, environmental and lifestyle risk factors, etc. Hypertension and hyperlipidemia were defined according to self-reported disease history. Current smokers were defined as those who had been smoking at least one cigarette per day for more than six months. Past smokers were those who smoked regularly in the past but had not smoked for at least one month. Never smokers were those who had never smoked or those who did not smoke as regularly as the current smokers. Current drinkers were defined as those who had been drinking at least 50 mL of liquor per week for at least six months. The definitions of past drinkers and never drinkers were similar to those of past smokers and never smokers, respectively.

The participants were required to fast overnight before blood sampling. Ten milliliter of venous blood samples was obtained for each participant, equally split into two tubes, with an anticoagulation and a coagulation accelerator, respectively. Serum glucose, hemoglobin A1c (HbA1c), and other serum indicators were analyzed in biochemical tests. For those fasting as required, serum glucose was considered as fasting blood glucose (FBG); for those not fasting, the serum glucose was considered as random blood glucose.

### 4.6. Statistical Analysis

Continuous variables were described as the means and standard deviations, and one-way analysis of variance (ANOVA) or Kruskal–Wallis test were applied to compare continuous variables. Categorical variables were described as proportions, and Pearson chi-squared test was used to compare different groups. For rs2925979, the genotype value was coded as the count of T alleles (i.e., an additive model). Obesity-related phenotypes were transformed by inverse standard normal function, unless indicated otherwise. To account for the correlation among family members, we applied logistic mixed effects models for the association analysis of T2DM as a binary outcome, and linear mixed effects models for obesity-related phenotypes as continuous outcomes, with each family as a stratum. For association analyses, we first analyzed all subjects, then males and females, separately. Sex–gene interactions were tested by including a cross-product of sex by rs2925979_T allele count in the regression models. For the association between rs2925979 and T2DM, we first analyzed without adjusting for covariates in Model 1. Then, we adjusted for age, hypertension, hyperlipidemia, smoking status, and alcohol drinking in Model 2. In the association analyses of rs2925979 with obesity-related phenotypes, we also adjusted for the covariates listed above. All statistical analyses were conducted by R (v.3.3.3). A two-sided *p* < 0.05 was considered as statistical significant.

## 5. Conclusions

In summary, *CMIP* rs2925979_T allele was associated with an increasing risk of T2DM and with decreasing levels of most obesity-related phenotypes in females, exerting pleiotropic genetic effects. Sex genetic architecture may influence the genetic effects of *CMIP* rs2925979 polymorphisms on T2DM and obesity.

## Figures and Tables

**Table 1:** Anthropometric characteristics of participants according to T2DM status.

Characteristics	Normal	Prediabetes	T2DM	*p-*Value
	(*n* = 899)	(*n* = 963)	(*n* = 1582)	
Age (years), mean (SD)	57.5 ± 8.2	59.4 ± 7.8	59.3 ± 7.5	4.04 × 10^−8^
Male, %	56.8	52.2	42.2	8.01 × 10^−13^
Hypertension, %	53.3	59.8	61.7	1.88 × 10^−4^
Hyperlipidemia, %	25.1	32.1	45.0	<2.20 × 10^−16^
Smoking status				5.00 × 10^−5^
Never smoker, %	50.3	51.6	58.4	
Past smoker, %	17.9	16.6	15.3	
Current smoker, %	31.8	31.9	26.3	
Alcohol drinking				3.90 × 10^−16^
Never drinker, %	53.4	53.8	66.9	
Past drinker, %	11.4	16.2	8.7	
Current drinker, %	35.2	30.0	24.4	
Rs2925979, %				0.261
TT genotype	14.8	14.8	17.2	
TC genotype	49.3	48.9	49.6	
CC genotype	36.0	36.2	33.1	

T2DM, type 2 diabetes mellitus; SD, standard deviation.

**Table 2:** Comparisons of the levels of obesity-related phenotypes between nondiabetes (prediabetes) and normal subjects, mean (SD).

Phenotype	Total			Male			Female		
	(*n* = 1862)			(*n* = 1014)			(*n* = 848)		
	Normal (*n* = 899)	Prediabetes (*n* = 963)	*p-*Value	Normal (*n* = 511)	Prediabetes (*n* = 503)	*p-*Value	Normal (*n* = 388)	Prediabetes (*n* = 460)	*p-*Value
BMI (kg/m^2^)	25.4 (3.4)	26.2 (3.5)	7.10 × 10^−7^ *	25.1 (3.2)	25.6 (3.3)	6.63 × 10^−4^ *	25.9 (3.5)	26.8 (3.7)	5.48 × 10^−4^ *
WC (cm)	89.5 (9.5)	91.4 (9.3)	2.30 × 10^−5^ *	90.2 (9.3)	91.8 (9.4)	8.45 × 10^−4^ *	88.6 (9.6)	90.9 (9.1)	0.015
HC (cm)	99.4 (7.5)	100.4 (7.5)	8.12 × 10^−4^ *	98.9 (6.8)	99.7 (7.0)	0.005 *	100.0 (8.3)	101.2 (7.9)	0.060
WHR	0.90 (0.06)	0.91 (0.05)	0.009	0.91 (0.05)	0.92 (0.05)	0.025	0.89 (0.06)	0.90 (0.05)	0.220
WHRadjBMI	−0.20 (1.01)	−0.15 (0.98)	0.472	−0.18 (1.00)	−0.14 (1.01)	0.618	−0.23 (1.03)	−0.17 (0.95)	0.656
PBF (%)	26.7 (9.2)	28.8 (9.6)	1.01 × 10^−6^ *	20.5 (5.5)	21.5 (5.6)	0.003 *	34.5 (6.6)	36.5 (6.5)	1.57 × 10^−4^ *
PBF of arms (%)	22.9 (10.1)	25.0 (10.8)	2.33 × 10^−6^ *	15.7 (4.3)	16.2 (4.2)	0.012	32.1 (7.7)	34.3 (7.5)	1.31 × 10^−4^ *
PBF of legs (%)	27.5 (8.9)	29.3 (9.2)	7.48 × 10^−7^ *	20.6 (4.2)	21.4 (4.4)	5.44 × 10^−4^ *	36.3 (4.7)	37.5 (4.6)	3.53 × 10^−4^ *
PBF of trunk (%)	26.7 (9.8)	29.2 (10.1)	7.39 × 10^−7^ *	21.2 (7.1)	22.6 (7.3)	0.002 *	33.7 (8.1)	36.2 (7.7)	1.01 × 10^−4^ *

BMI, body mass index; WC, waist circumference; HC, hip circumference; WHR, waist-to-hip ratio; WHRadjBMI, waist-to-hip ratio adjusted for body mass index; PBF, percentage of body fat; BMI, WC, HC, WHR, and PBF-related phenotypes (termed as “PBFs” in the following context) are displayed as original values, not transformed by the inverse standard normal function; *p* values for results of linear mixed effects regression models adjusted for age, hypertension, hyperlipidemia, smoking status, and alcohol drinking; *, significant after Bonferroni correction (*p* < 0.006).

**Table 3:** Association between *CMIP* rs2925979_T allele numbers and T2DM.

	Total		Male		Female		*p-*Value for Sex Interaction
	(*n* = 3444)		(*n* = 1681)		(*n* = 1763)		
	*OR* (95%*CI*)	*p-*Value	*OR* (95%*CI*)	*p-*Value	*OR* (95%*CI*)	*p-*Value	
Model 1	1.15 (1.02~1.30)	0.022	0.96 (0.79~1.17)	0.705	1.29 (1.11~1.50)	9.30 × 10^−4^	0.021
Model 2	1.17 (1.03~1.32)	0.014	0.98 (0.80~1.20)	0.809	1.34 (1.14~1.58)	4.70 × 10^−4^	0.013

T2DM, type 2 diabetes mellitus; *OR*, odds ratio; *CI*, confidence interval; Model 1, not adjusted; Model 2, adjusted for age, hypertension, hyperlipidemia, smoking status, and alcohol drinking.

**Table 4:** Association between *CMIP* rs2925979_T allele numbers and obesity-related phenotypes among nondiabetic subjects.

Phenotype	Total		Male		Female		*p-*Value for Sex Interaction
	(*n* = 1862)		(*n* = 1014)		(*n* = 848)		
	*β* (*SE*)	*p-*Value	*β* (*SE*)	*p-*Value	*β* (*SE*)	*p-*Value	
BMI	−0.079 (0.034)	0.019	−0.015 (0.044)	0.734	−0.161 (0.052)	0.002 *	0.037
WC	−0.055 (0.035)	0.111	−0.005 (0.045)	0.914	−0.136 (0.052)	0.010	0.083
HC	−0.073 (0.035)	0.040	−0.000 (0.046)	0.995	−0.175 (0.054)	0.001 *	0.016
WHR	−0.017 (0.036)	0.623	−0.011 (0.047)	0.820	−0.030 (0.053)	0.571	0.963
WHRadjBMI	0.034 (0.036)	0.354	0.015 (0.049)	0.765	0.050 (0.055)	0.357	0.626
PBF	−0.067 (0.035)	0.056	−0.002 (0.046)	0.974	−0.149 (0.052)	0.004 *	0.035
PBF of arms	−0.045 (0.035)	0.202	0.023 (0.046)	0.622	−0.130 (0.053)	0.014	0.027
PBF of legs	−0.073 (0.035)	0.039	−0.010 (0.047)	0.827	−0.152 (0.053)	0.004 *	0.040
PBF of trunk	−0.077 (0.035)	0.029	−0.016 (0.046)	0.730	−0.153 (0.053)	0.004 *	0.054

All obesity-related phenotypes were transferred by the inverse standard normal function; results adjusted for age, hypertension, hyperlipidemia, smoking status, and alcohol drinking; *, significant after Bonferroni correction (*p* < 0.006); Abbreviations as in Table 2.

## Supplementary Materials

Supplementary materials can be found at http://www.mdpi.com/1422-0067/19/4/1011/s1.

## Abbreviations

CMIP	C-maf-Inducing Protein
T2DM	Type 2 diabetes mellitus
BMI	Body mass index
WC	Waist circumference
HC	Hip circumference
PBF	Percentage of body fat
GWAS	Genome-wide association study
NF-κB	Nuclear factor-κB
Th2	T-helper 2
SNP	Single nucleotide polymorphism
WHR	Waist-to-hip ratio
WHRadjBMI	Waist-to-hip ratio adjusted for body mass index
*OR*	Odds ratio
95% *CI*	95% confidence interval
*SD*	Standard deviation
*SE*	Standard error
*CDKN2A/2B*	Cyclin-Dependent Kinase Inhibitor 2A/2B
*KCNJ11*	Potassium Voltage-Gated Channel Subfamily J Member 11
*TCF7L2*	Transcription Factor 7 Like 2
*ELMO1*	Engulfment and Cell Motility 1
*BCL11A*	B Cell CLL/Lymphoma 11A
DHEAS	Dihydroepiandrosterone sulphate
*ARL15*	ADP Ribosylation Factor Like GTPase 15
*QPCTL*	Glutaminyl-Peptide Cyclotransferase-Like
*CPEB4*	Cytoplasmic Polyadenylation Element Binding Protein 4
