# Supplementary material for: Opposite Genetic Effects of CMIP Polymorphisms on the Risk of Type 2 Diabetes and Obesity: A Family-Based Study in China
Source: Int J Mol Sci. 2018 Mar 28;19(4):1011. doi: 10.3390/ijms19041011 (PMC5979311; doi:10.3390/ijms19041011)
Supplement: Supplementary file 1 [file ijms-19-01011-s001.pdf]

International Journal of
Molecular Sciences

MDPI

Table S1. Pearson correlation coefficients among obesity-related phenotypes.

	BMI	WC	HC	WHR	WHRadjBMI	PBF	PBF of Arms	PBF of Legs	PBF of Trunk
**BMI**	1.000	0.812	0.823	0.391	−0.014	0.666	0.616	0.531	0.732
**WC**	0.812	1.000	0.823	0.713	0.361	0.458	0.383	0.307	0.546
**HC**	0.823	0.823	1.000	0.190	−0.179	0.542	0.493	0.410	0.610
**WHR**	0.391	0.713	0.190	1.000	0.849	0.132	0.065	0.034	0.200
**WHRadjBMI**	−0.014	0.361	−0.179	0.849	1.000	0.027	0.005	0.013	0.039
**PBF**	0.666	0.458	0.542	0.132	0.027	1.000	0.977	0.965	0.983
**PBF of arms**	0.616	0.383	0.493	0.065	0.005	0.977	1.000	0.966	0.937
**PBF of legs**	0.531	0.307	0.410	0.034	0.013	0.965	0.966	1.000	0.903
**PBF of trunk**	0.732	0.546	0.610	0.200	0.039	0.983	0.937	0.903	1.000

Abbreviations as in Table 2.

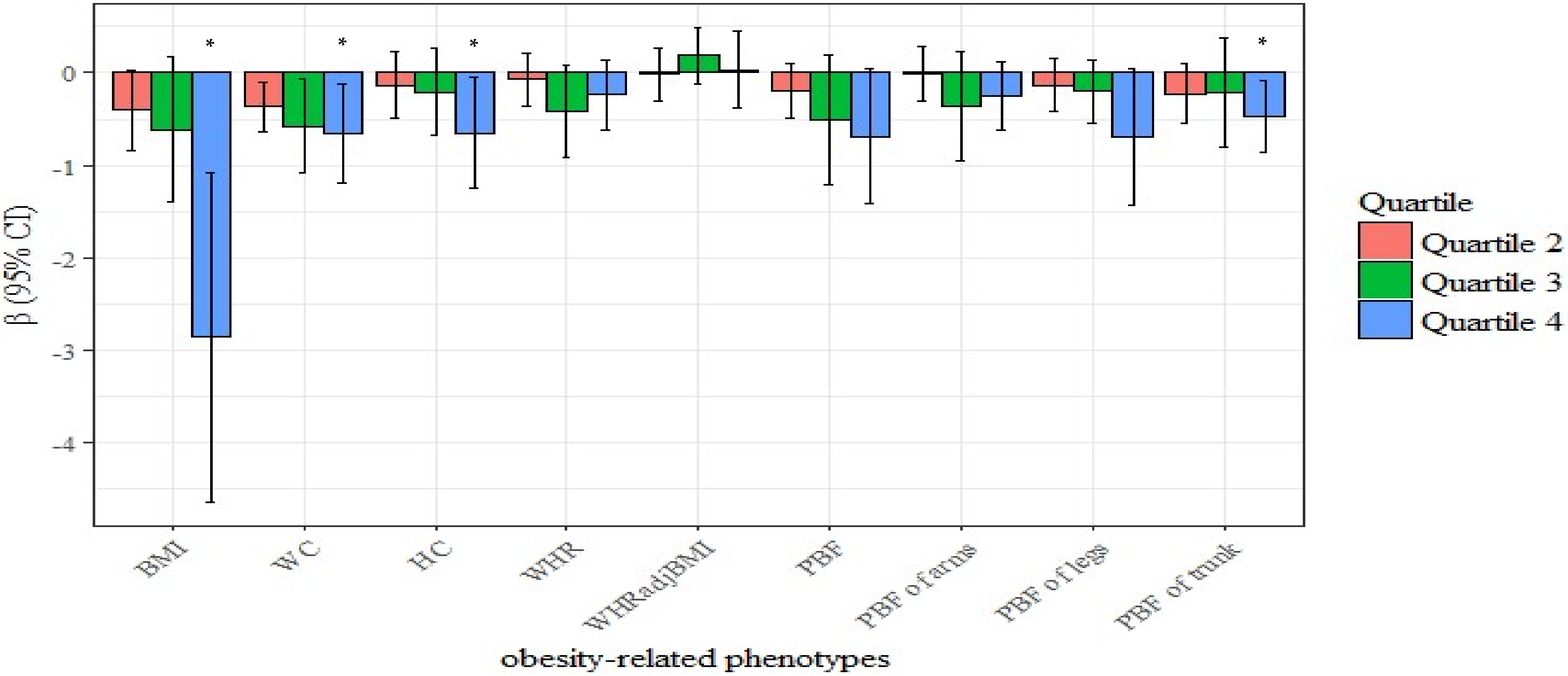

Figure S1. Association between rs2925979_T allele number and obesity-related phenotypes (stratified by quartiles of obesity-related phenotypes). Results of association analyses adjusted for age, hypertension, hyperlipidemia, smoking status, and alcohol drinking; *, $p < 0.05$; abbreviations as in Table 2.

Table S2. Association between *CMIP* rs2925979_T allele numbers and obesity-related phenotypes among T2DM patients.

	Combined (n = 1582)		Male (n = 667)		Female (n = 915)		p-Value for Sex Interaction
	β (SE)	p-Value	β (SE)	p-Value	β (SE)	p-Value	
BMI	−0.026 (0.036)	0.482	0.023 (0.058)	0.695	−0.070 (0.046)	0.128	0.332
WC	0.010 (0.037)	0.790	0.081 (0.057)	0.155	−0.045 (0.047)	0.336	0.092
HC	−0.051 (0.037)	0.169	0.006 (0.059)	0.913	−0.109 (0.048)	0.022	0.088
WHR	0.109 (0.037)	0.003*	0.146 (0.057)	0.010	0.086 (0.047)	0.068	0.386
WHRadjBMI	0.121 (0.037)	0.001*	0.128 (0.056)	0.022	0.122 (0.049)	0.013	0.805
PBF	−0.023 (0.037)	0.533	0.051 (0.060)	0.392	−0.079 (0.048)	0.097	0.125
PBF of arms	−0.016 (0.037)	0.668	0.064 (0.059)	0.271	−0.073 (0.047)	0.122	0.103
PBF of legs	−0.008 (0.037)	0.837	0.066 (0.058)	0.256	−0.059 (0.047)	0.213	0.154
PBF of trunk	−0.032 (0.037)	0.380	0.046 (0.059)	0.436	−0.089 (0.047)	0.059	0.094

All obesity-related phenotypes were transferred by the inverse standard normal function; *, significant after Bonferroni correction ($p < 0.006$); abbreviations as in Table 2.

Table S3. Inclusion and exclusion criteria for participants.

**(a) Inclusion criteria for T2DM group**
1. With a history of T2DM or
2. With HbA1c ≥ 6.5%, or FBG (fasting blood glucose) ≥ 7.0 mmol/L, or random blood glucose ≥ 11.1 mmol/L
**(b) Inclusion criteria for prediabetes group**
1. Without any type of diabetes and
2. With 5.7% ≤ HbA1c < 6.5% or 5.6 mmol/L ≤ FBG < 7.0 mmol/L
**(c) Inclusion criteria for normal group**
1. Without any type of diabetes and
2. With HbA1c < 5.7% and FBG < 5.6 mmol/L
**(d) Exclusion criteria for all participants**
1.With a history of type 1 diabetes, gestational diabetes, or any other type of diabetes except T2DM
2. With an onset age of diabetes < 30 years old
3. Diagnosed with diabetic ketoacidosis on the early stage of diabetes
4. With serious diseases or diabetic complications, not able to coordinate with the study

T2DM, type 2 diabetes mellitus; HbA1c, hemoglobin a1c; FBG, fasting blood glucose.
